# The Linear ubiquitin chain assembly complex acts as a liver tumor suppressor and inhibits hepatocyte apoptosis and hepatitis

**DOI:** 10.1002/hep.29074

**Published:** 2017-04-10

**Authors:** Yutaka Shimizu, Nieves Peltzer, Alexandra Sevko, Elodie Lafont, Aida Sarr, Helena Draberova, Henning Walczak

**Affiliations:** ^1^ Centre for Cell Death, Cancer and Inflammation, UCL Cancer InstituteUniversity College LondonLondonUK

## Abstract

Linear ubiquitination is a key posttranslational modification that regulates immune signaling and cell death pathways, notably tumor necrosis factor receptor 1 (TNFR1) signaling. The only known enzyme complex capable of forming linear ubiquitin chains under native conditions to date is the linear ubiquitin chain assembly complex, of which the catalytic core component is heme‐oxidized iron regulatory protein 2 ubiquitin ligase‐1–interacting protein (HOIP). To understand the underlying mechanisms of maintenance of liver homeostasis and the role of linear ubiquitination specifically in liver parenchymal cells, we investigated the physiological role of HOIP in the liver parenchyma. To do so, we created mice harboring liver parenchymal cell–specific deletion of HOIP (*Hoip^Δhep^* mice) by crossing *Hoip*‐floxed mice with albumin–Cre mice. HOIP deficiency in liver parenchymal cells triggered tumorigenesis at 18 months of age preceded by spontaneous hepatocyte apoptosis and liver inflammation within the first month of life. In line with the emergence of inflammation, *Hoip^Δhep^* mice displayed enhanced liver regeneration and DNA damage. In addition, consistent with increased apoptosis, HOIP‐deficient hepatocytes showed enhanced caspase activation and endogenous formation of a death‐inducing signaling complex which activated caspase‐8. Unexpectedly, exacerbated caspase activation and apoptosis were not dependent on TNFR1, whereas ensuing liver inflammation and tumorigenesis were promoted by TNFR1 signaling. *Conclusion*: The linear ubiquitin chain assembly complex serves as a previously undescribed tumor suppressor in the liver, restraining TNFR1‐independent apoptosis in hepatocytes which, in its absence, is causative of TNFR1‐mediated inflammation, resulting in hepatocarcinogenesis. (Hepatology 2017;65:1963‐1978).

**Abbreviations**
Alb‐Cre: albumin promoter–driven Cre recombinase
ALT: alanine aminotransferase
CC3: cleaved caspase 3
Ccl3: chemokine (C‐C motif) ligand 3
CD: cluster of differentiation
CD95L: CD95 ligand
c‐FLIP_L_: cellular FLICE‐like inhibitory protein long isoform
CHX: cycloheximide
cpdm: chronic proliferative dermatitis mice
Cre: cyclization recombination
Cxcl1: chemokine (C‐X‐C motif) ligand 1
DD: death domain
ERK: extracellular signal‐regulated kinase
FADD: Fas‐associated with DD
HCC: hepatocellular carcinoma
HOIL‐1: heme‐oxidized iron regulatory protein 2 ubiquitin ligase
HOIP: heme‐oxidized iron regulatory protein 2 ubiquitin ligase–interacting protein
IKK: IκB kinase
JNK: c‐Jun N‐terminal kinase
KO: knockout
LPC: liver parenchymal cell
LUBAC: linear ubiquitin chain assembly complex
NEMO: nuclear factor‐κB essential modulator
NF‐κB: nuclear factor‐κB
poly(I:C): polyinosinic:polycytidylic acid
RIPK: receptor‐interacting protein kinase
SHARPIN: SHANK‐associated RH domain interacting protein
TAK1: transforming growth factor β–activated kinase 1
TNF: tumor necrosis factor
TNFR1: tumor necrosis factor receptor 1
TRAIL: TNF‐related apoptosis‐inducing ligand
TUNEL: terminal deoxynucleotidyl transferase–mediated deoxyuridine triphosphate nick‐end labeling

Chronic hepatitis is the leading cause of liver cancer, which is one of the most lethal cancers worldwide.^1^, ^2^ Infection with hepatotropic viruses and excessive alcohol intake are among the major causes of hepatitis. As tumor necrosis factor (TNF) is a major proinflammatory cytokine which can induce liver cell apoptosis,^3^ TNF blockade has been tested in patients suffering from hepatic complications. Indeed, therapies targeting TNF have shown promising results in improving nonalcoholic steatohepatitis in humans.^4^, ^5^ However, by contrast, anti‐TNF therapy has not been successful in ameliorating alcoholic hepatitis^6^ and, unexpectedly, can even trigger reactivation of hepatitis B virus infection by preventing viral clearance.^7^ Thus, dissection of the TNF/TNF receptor 1 (TNFR1) system in distinct etiologies and cell types is crucial to improve the strategy for intervention of liver diseases.

Several studies have revealed a key role for TNFR1 signaling in liver inflammation and cancer in mouse models. For instance, liver parenchymal cell (LPC)–specific IκB kinase (IKK) β–deficient mice are more susceptible to diethylnitrosamine‐induced cell death and carcinogenesis.^8^ Follow‐up studies employing LPC‐specific nuclear factor‐κB (NF‐κB) essential modulator (NEMO) and transforming growth factor β–activated kinase 1 (TAK1) knockout (KO) mice indicated that indeed optimal regulation of TNFR1 signaling is crucial to maintain liver homeostasis and to protect from inflammation and cancer.^9^, ^10^ Importantly, in addition to the regulation of NF‐κB, aberrant activation of cell death machineries triggered by deregulated TNFR1 signaling can evoke severe liver pathology. For instance, hepatocyte apoptosis and hepatocarcinogenesis due to LPC‐specific TAK1 deficiency are mediated by caspase‐8 in those cells, while cholestasis was induced by the necroptosis inducer receptor–interacting protein kinase (RIPK3).^11^ Furthermore, transforming growth factor β–activated kinase 1 and the IKKα–IKKβ–NEMO complex have been shown to suppress cell death signaling independently of their role in activating NF‐κB in hepatocytes and other cell types.^12^, ^13^ Thus, deregulated cell death and/or NF‐κB signaling in hepatocytes can result in liver inflammation and carcinogenesis.

Ubiquitination is a crucial posttranslational modification mediated by ubiquitin ligases to define and fine‐tune the biological function of substrate proteins.^14^ Notably, the linear ubiquitin chain assembly complex (LUBAC), the only known ubiquitin ligase complex capable of forming linear diubiquitin linkages *de novo*,^15^ has pivotal immunoregulatory and anti–cell death functions in a wide range of innate and adaptive immune signaling pathways, including those triggered by TNFR1.^16^, ^17^, ^18^ LUBAC is comprised of heme‐oxidized iron regulatory protein 2 ubiquitin ligase–interacting protein (HOIP), heme‐oxidized iron regulatory protein 2 ubiquitin ligase (HOIL‐1), and SHANK‐associated RH domain interacting protein (SHARPIN).^15^, ^19^ Deficiency in HOIP, the catalytic LUBAC component, results in embryonic lethality at mid‐gestation due to TNFR1‐mediated endothelial cell death.^20^ Indeed, HOIP‐deficient mouse embryonic fibroblasts display a robust formation of the cell death–inducing signaling platform complex II of TNFR1 signaling, containing Fas‐associated with death domain (FADD), caspase‐8, RIPK1, and RIPK3, upon TNF stimulation.^20^ SHARPIN‐null mice (chronic proliferative dermatitis mice or *cpdm* mice), by contrast, develop TNFR1‐dependent multiorgan inflammation, including the liver, with TNFR1‐independent aberration in lymphoid tissues.^19^, ^21^, ^22^ Interestingly, abnormalities in *cpdm* mice are completely corrected by genetic ablation of RIPK3 and heterozygosity of caspase‐8 or epidermal ablation of FADD.^21^, ^23^ These studies collectively corroborate a central role of LUBAC in restraining aberrant activation of TNFR1‐induced cell death machineries in order to maintain tissue homeostasis.

Although *cpdm* mice exhibit liver inflammation, it remains unknown which tissues and cell types contribute to hepatitis. In addition, the physiological role of LUBAC in LPCs remains unknown. Here, we investigated the role of linear ubiquitination and LUBAC in liver inflammation and carcinogenesis by studying mice that lack HOIP, the central and catalytically active component of LUBAC, specifically in LPCs.

## Materials and Methods

### ANIMALS

All animal studies were conducted according to an appropriate license under the Animals (Scientific Procedures) Act of 1986. HOIP‐floxed (*Hoip^flox^*) mice were created from the C57BL/6 embryonic stem cell clone EPD0161_5_A05 from KOMP Repository (http://www.komp.org) and generated by the Wellcome Trust Sanger Institute (Hinxton, UK).^20^ *Hoip^flox^* mice were subsequently crossed to albumin promoter–driven Cre recombinase (*Alb‐Cre*) mice (B6.Cg‐Tg[Alb‐cre]21Mgn/J) purchased from Jackson Laboratories (Bar Harbor, ME) to delete HOIP in LPCs. TNFR1‐deficient mice (B6.129‐Tnfrsf1atm1Mak/J) and cluster of differentiation 95 (CD95) death domain (DD)–floxed mice (C57BL/6‐Fastm1Cgn/J) were purchased from Jackson Laboratories. Mice were maintained at Charles River (Margate, UK) and UCL Biological Service Unit (London, UK) under specific pathogen‐free conditions and a 12‐hour dark/light cycle and fed *ad libitum*. Both males and females were used in the experiments.

### OTHER METHODS

For details of histology and immunohistochemistry, immunoprecipitation, flow cytometric analysis of liver immune cells, isolation of mouse primary hepatocytes, cell viability assay, western blotting, tissue RNA extraction, quantitative RT‐PCR, and RNA sequencing, see Supporting Information.

## Results

### HEPATOCYTE‐SPECIFIC DELETION OF HOIP LEADS TO LIVER TUMORIGENESIS

To specifically investigate the role of linear ubiquitination in the maintenance of liver homeostasis, we generated mice with LPC‐specific deletion of HOIP by crossing *Hoip^flox^* mice^20^ with *Alb‐Cre* mice.^24^ Mice deficient for HOIP in the liver, referred to as *Hoip^Δhep^* mice, showed efficient ablation of HOIP protein in primary hepatocytes at 8‐9 weeks of age (Supporting Fig. S1A). The levels of the other two LUBAC components, HOIL‐1 and SHARPIN, were mildly reduced by abrogation of HOIP, in line with previous reports on other tissues and cells.^19^, ^20^, ^25^ TNFR signaling complex pull‐down analysis revealed that HOIP‐deficient cells produced drastically reduced levels of linear ubiquitination within the TNFR signaling complex (Supporting Fig. S1B). The residual linear ubiquitination observed in hepatocytes isolated from *Hoip^Δhep^* mice is most likely due to an incomplete penetrance of gene deletion by Alb‐Cre, which can be seen in substantially reduced yet detectable levels of HOIP in these cells.

**Figure 1:**
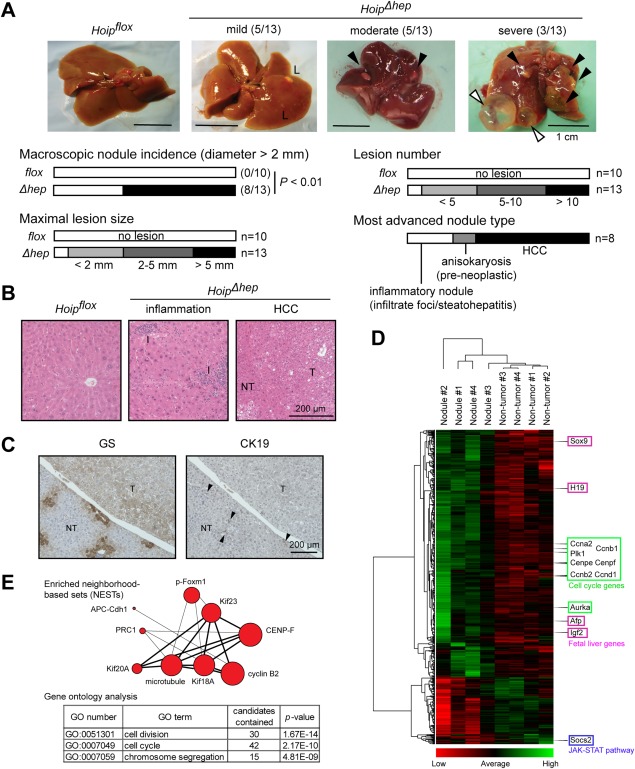
HOIP deletion leads to spontaneous liver tumorigenesis. (A) Representative pictures of livers from *Hoip^flox^* and *Hoip^Δhep^* mice at 18 months of age (upper panels). Black arrowheads indicate large nodules, and white arrowheads indicate cystic lesions. Bar graphs indicate the incidence of macroscopic nodules (diameter >2 mm), maximal lesion size, lesion number, and the most advanced tumor type in *Hoip^Δhep^* livers with macroscopic nodules (diameter >2 mm). Fisher's exact test was employed for the statistical analysis of incidence of macroscopic nodules. (B) Hematoxylin and eosin staining of *Hoip^flox^* liver and lesion areas (inflamed and HCC) in *Hoip^Δhep^* livers. (C) Glutamine synthase and cytokeratin 19 staining of nontumoral and tumoral areas of the liver in *Hoip^Δhep^* mice. Arrowheads indicate cytokeratin 19–positive bile ducts. (D) Clustering analysis of 714 differentially expressed genes (*q* value < 0.05) in the nodules in *Hoip^Δhep^* livers compared to nontumor samples in these livers. (E) Upper schematics represent enriched neighborhood‐based sets of the top 182 differentially expressed genes in the tumor nodules (log_2_ [tumor/nontumor] >1.2) using ConsensusPathDB. The size of circles corresponds to the number of related genes found in the analyzed gene set. More than five genes shared in the two nodes are connected with a line (thicker line, 10 or more genes shared). Lower tables show enriched gene ontology–based sets of the 182 differentially expressed genes. Abbreviations: CK19, cytokeratin 19; GO, gene ontology; GS, glutamine synthase; L, lesional area; NT, nontumor; T, tumor.

*Hoip^Δhep^* mice were as viable as *Hoip^flox^* littermate controls at least up to 18 months (Supporting Fig. S1C). However, at this stage the vast majority of *Hoip^Δhep^* mice developed macroscopic lesions and nodules in the liver, while age‐matched littermate control mice did not show any overt liver pathology (Fig. 1A, top panels). The size, number, and severity of macroscopic lesions appearing in *Hoip^Δhep^* livers were variable, with some mice developing mild (small lesions; 5/13), moderate (multiple lesions and nodules; 5/13), or severe (large nodules and cystic lesions; 3/13) pathology (Fig. 1A; Supporting Fig. S2A,B). Histopathological analysis showed that more than half of the *Hoip^Δhep^* animals displaying moderate or severe pathology developed hepatocellular carcinoma (HCC) (5/8) and that those which had not developed HCC displayed precancerous anisokaryosis or inflammatory foci (Fig. 1A,B). The tumor nodules analyzed stained positively for glutamine synthase and were negative for cytokeratin 19, indicating that the tumors originated from the hepatocyte and not the cholangiocyte lineage (Fig. 1C; Supporting Fig. S2C). Of note, glutamine synthase staining showed a diffuse pattern, which is often observed in human HCC.^26^ In addition, *Hoip^Δhep^* livers displayed focal lipid accumulation, which was occasionally accompanied by inflammation, indicating that *Hoip^Δhep^* mice developed steatosis (Supporting Fig. S2D).

In order to further molecularly characterize the tumors arising in *Hoip^Δhep^* mice, we used RNA sequencing to compare the gene expression profiles of nontumor tissue to tumor nodules from *Hoip^Δhep^* mice. The expression profiles of all four nodular samples analyzed were clearly distinguished from those of nontumorous samples (Fig. 1D). The genes which were up‐regulated in the nodular samples were predominantly regulators of mitosis and cell cycle progression (Fig. 1E). This expression signature observed in the tumor samples from *Hoip^Δhep^* mice resembles that of the subclass A and G1‐G3 of human HCC described by Thorgeirsson's and Zucman‐Rossi's groups, respectively, which are correlated with poor prognosis.^27^, ^28^ Thus, HOIP deletion results in late formation of hepatic tumor nodules with overexpressed cell cycle regulatory genes.

### INFLAMMATION ACCOMPANIED BY DNA DAMAGE EMERGES AT EARLY STAGES OF LIFE IN HOIP‐DEFICIENT LIVERS

**Figure 2:**
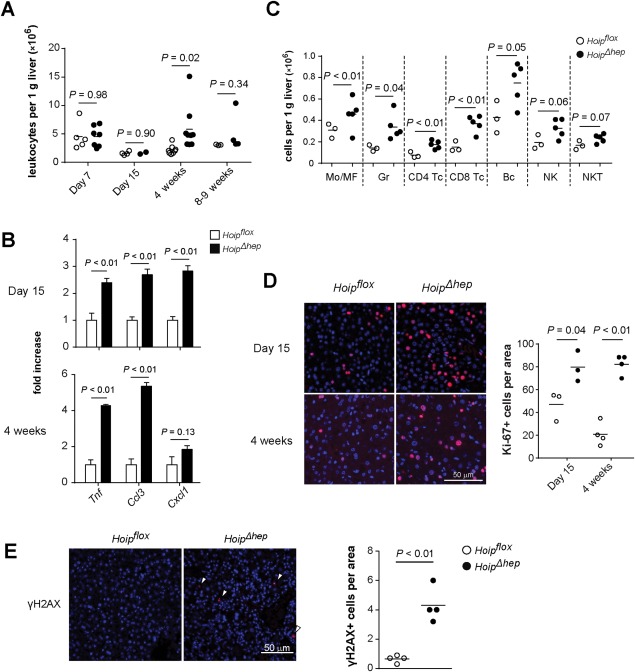
Inflammation accompanied by DNA damage emerges in HOIP‐deleted livers. (A) Hepatic CD45^+^ cell count per gram of *Hoip^flox^* (open circles) and *Hoip^Δhep^* mice (closed circles) at the indicated ages. (B) Relative quantification of the transcript levels of *Tnf*, *Ccl3*, and (*Cxcl1*) in *Hoip^flox^* and *Hoip^Δhep^* livers (n = 4 each). Levels of transcripts were normalized to those of *Hoip^flox^* livers. (C) Liver cells from *Hoip^flox^* and *Hoip^Δhep^* mice at 4 weeks of age were stained, and the numbers of hepatic immune subpopulations were quantified by flow cytometry. (D) Ki‐67 staining (red) of liver sections *from Hoip^flox^* and *Hoip^Δhep^* mice at 4 weeks of age. The dot plot shows the quantification of Ki‐67^+^ cells per optical area. (E) γH2AX staining (red) of liver sections from *Hoip^flox^* and *Hoip^Δhep^* mice at 4 weeks of age. The dot plots show the quantification of γH2AX^+^ cells per optical area. Unpaired two‐tailed *t* test was employed for statistical analysis. Abbreviations: Bc, B220^+^ cells; CD4 Tc, CD3^+^ CD4^+^ cells; CD8 Tc, CD3^+^ CD8^+^ cells; Gr, Ly6G^+^ cells; Mo/MF, F4/80^+^ cells; NK, CD3^−^ NK1.1^+^ natural killer cells; NKT, CD3^+^ NK1.1^+^ natural killer T cells.

Inflammation is often a crucial step in liver carcinogenesis. In order to decipher whether deletion of HOIP from hepatocytes triggered inflammation, we studied *Hoip^Δhep^* livers within the first months of life. We first tracked the number of leukocytes in the liver by counting hepatic CD45^+^ cells from *Hoip^flox^* and *Hoip^Δhep^* animals from postnatal day 7 up to 8‐9 weeks of age. At postnatal days 7 and 15, leukocyte counts were comparable between *Hoip^flox^* and *Hoip^Δhep^* livers (Fig. 2A). Yet, at 4 weeks of age, leukocyte numbers significantly increased in *Hoip^Δhep^* livers compared to control livers (Fig. 2A). In accord, elevated levels of *Tnf*, chemokine (C‐C motif) ligand 3 (*Ccl3*), and chemokine (C‐X‐C motif) ligand 1 (*Cxcl1*) were observed at 4 weeks and notably at 2 weeks of age, when inflammation was not detected in *Hoip^Δhep^* livers by leukocyte count (Fig. 2B). However, the inflammation observed at 4 weeks was transient as the difference in leukocyte numbers between *Hoip^flox^* and *Hoip^Δhep^* became marginal at 8‐9 weeks of age (Fig. 2A). Infiltration of myeloid cells was observed at the sites of inflammation in *Hoip^Δhep^* livers at 4 weeks of age (Supporting Fig. S3). To further investigate whether certain immune cell compartments were particularly involved in the onset of inflammation in *Hoip^Δhep^* livers at 4 weeks, we analyzed the various subpopulations of liver cells by flow cytometry. Monocytes/macrophages, granulocytes, CD4^+^ and CD8^+^ T cells, and B cells were accumulated in *Hoip^Δhep^* livers compared to control livers (Fig. 2C). This result showed that immune cells from both the myeloid and lymphoid compartments are recruited to the inflamed *Hoip^Δhep^* livers and participate in inflammatory responses, which coincides with increased production of cytokines. Together, these data indicate that deficiency of HOIP from liver parenchyma elicits an acute immune response in the first 4 weeks of life, followed by a resolution phase between weeks 4 and 9.

Upon liver damage, hepatocytes and liver cell progenitors undergo compensatory proliferation to replenish liver and maintain tissue integrity.^29^ Indeed, HOIP deletion resulted in initiation of liver regeneration as a higher number of Ki‐67^+^ proliferating liver cells in *Hoip^Δhep^* mice compared to littermate controls was detected at 2‐4 weeks (Fig. 2D). Additionally, an inflammatory environment can lead to accumulation of DNA damage and inflammation‐associated carcinogenesis.^30^ Intriguingly, *Hoip^Δhep^* livers presented increased γH2AX^+^ foci, which are used as a DNA damage marker (Fig. 2E). Taken together, HOIP deficiency in LPCs results in transient liver inflammation at early stages of life, and this inflammation is accompanied by a DNA damage response and regeneration of hepatocytes.

### HOIP DELETION IN THE LIVER PARENCHYMA RESULTS IN LIVER DAMAGE AND HEPATOCYTE APOPTOSIS PRIOR TO INFLAMMATION

**Figure 3:**
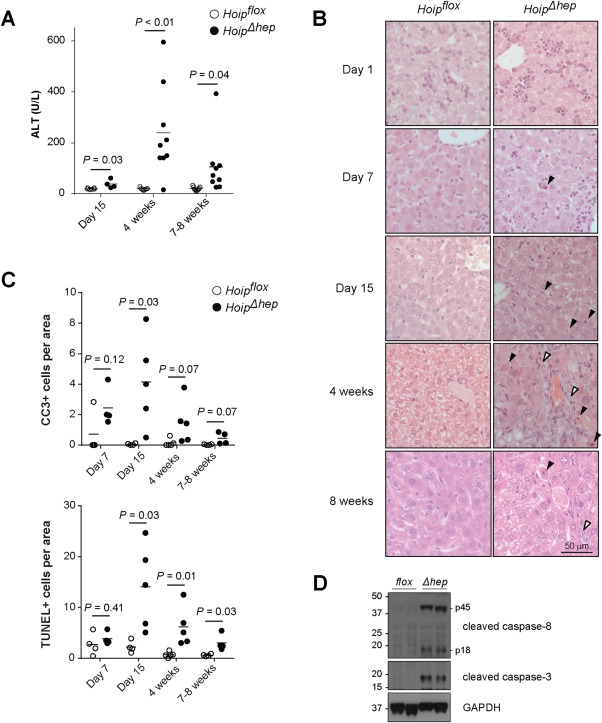
HOIP deletion in the liver parenchyma results in increased apoptosis. (A) Levels of serum ALT in *Hoip^flox^* (open circles) and *Hoip^Δhep^* mice (closed circles) at the indicated ages. (B) Hematoxylin and eosin staining of the livers of *Hoip^flox^* and *Hoip^Δhep^* mice at the indicated ages. Black arrowheads indicate apoptotic bodies, and white arrowheads indicate areas showing the ductular reaction. (C) Quantification of CC3^+^ and TUNEL^+^ cells per field of *Hoip^flox^* and *Hoip^Δhep^* liver sections. (D) Lysates of primary hepatocytes from *Hoip^flox^* and *Hoip^Δhep^* mice at 8‐10 weeks of age were immunoblotted for CC8 and CC3. Unpaired two‐tailed *t* test was employed for statistical analysis. Abbreviation: GAPDH, glyceraldehyde 3‐phosphate dehydrogenase.

To quantitatively evaluate the extent of liver damage in *Hoip^Δhep^* mice, we determined serum alanine aminotransferase (ALT) levels. In agreement with the leukocyte counts, serum levels of ALT from *Hoip^Δhep^* mice showed a moderate yet significant surge at 2 weeks, which peaked at 4 weeks and declined between 4 and 8 weeks of age (Fig. 3A). It is known that the *Alb‐Cre* mice express the Cre recombinase in cholangiocytes (bile duct cells) in addition to hepatocytes.^9^ Indeed, values of the bile duct damage marker alkaline phosphatase were significantly elevated in the sera of *Hoip^Δhep^* mice and correlated well with the surge in ALT values (Supporting Fig. S4A, left panel). A minority of *Hoip^Δhep^* mice also displayed high levels of serum bilirubin (>0.5 mg/dL), indicating that the animals suffered from cholestasis, which may contribute to liver damage (Supporting Fig. S4A, right panel). Collectively, these results show that HOIP deletion in LPCs elicits liver damage and hepatocyte death and, importantly, that both of these events predate the onset of inflammation.

We next aimed to identify the event that resulted in inflammation in *Hoip^Δhep^* livers at 4 weeks of age. To do so, we performed a histological analysis of *Hoip^Δhep^* mice from postnatal day 1 up to 8 weeks of age (Fig. 3B). While no aberration in *Hoip^Δhep^* livers was observed at postnatal day 1, apoptotic bodies in *Hoip^Δhep^* livers were detected at postnatal day 7, and they became more prevalent at 2 weeks of age compared to littermate controls. At 4 weeks of age, i.e., at the peak of inflammation (Fig. 2A), we detected tissue collapse in addition to apoptotic bodies and ductular reactions (Fig. 3B). In line with the observation that inflammation was ameliorated at 8 weeks of age (Fig. 2A), apoptotic bodies and ductular reactions were also attenuated in *Hoip^Δhep^* livers at this stage (Fig. 3B). Hence, increased cell death appeared to be the first pathological event in HOIP‐deficient livers.

To better characterize the type of cell death observed in the absence of HOIP from LPCs, we performed terminal deoxynucleotidyl transferase–mediated deoxyuridine triphosphate nick‐end labeling (TUNEL) and cleaved caspase‐3 (CC3) stainings. Both TUNEL^+^ and apoptotic CC3^+^ cells were significantly increased at 2, 4, and 8 weeks of age in *Hoip^Δhep^* livers (Fig. 3C; Supporting Fig. S4B). Notably, the number of dead cells was moderately elevated already during the first week of life in *Hoip^Δhep^* livers (Fig. 3C). The numbers of both TUNEL^+^ and CC3^+^ cells per area peaked at 2 weeks of age, thereby preceding the peak of liver leukocyte count and serum ALT levels by 2 weeks (Figs. 2A and 3A). To evaluate whether caspase‐3 activation could be a consequence of caspase‐8 cleavage, lysates from *Hoip^flox^* and *Hoip^Δhep^* livers were analyzed by western blotting. Caspase‐8 cleavage was indeed elevated in the livers of *Hoip^Δhep^* mice at 4 weeks of age (Supporting Fig. S4C). In accord, primary hepatocytes from *Hoip^Δhep^* mice displayed activation of caspase‐3 as well as caspase‐8 cleavage in their lysates (Fig. 3D). No obvious differences in the levels of any of the proapoptotic and antiapoptotic proteins evaluated in HOIP‐deficient hepatocytes was observed (Supporting Fig. S4D). Of note, we detected increased phosphorylation of IκBα and c‐Jun N‐terminal kinase (JNK), accompanied by a reduction in the levels of IκBα in *Hoip^Δhep^* livers, which is likely a consequence of the ongoing inflammation in these livers (Supporting Fig. S4E). Taken together, *Hoip^Δhep^* livers exhibit increased hepatocyte death, characterized by prominent activation of caspase‐8 and caspase‐3 as well as TUNEL positivity. Hence, mice with HOIP deficiency in the liver suffer from hepatocyte apoptosis which precedes the emergence of hepatitis.

### HOIP‐DEFICIENT HEPATOCYTES ARE MORE SENSITIVE TO APOPTOSIS INDUCTION BUT LESS CAPABLE OF NF‐κB ACTIVATION BY VARIOUS IMMUNE RECEPTOR STIMULI

**Figure 4:**
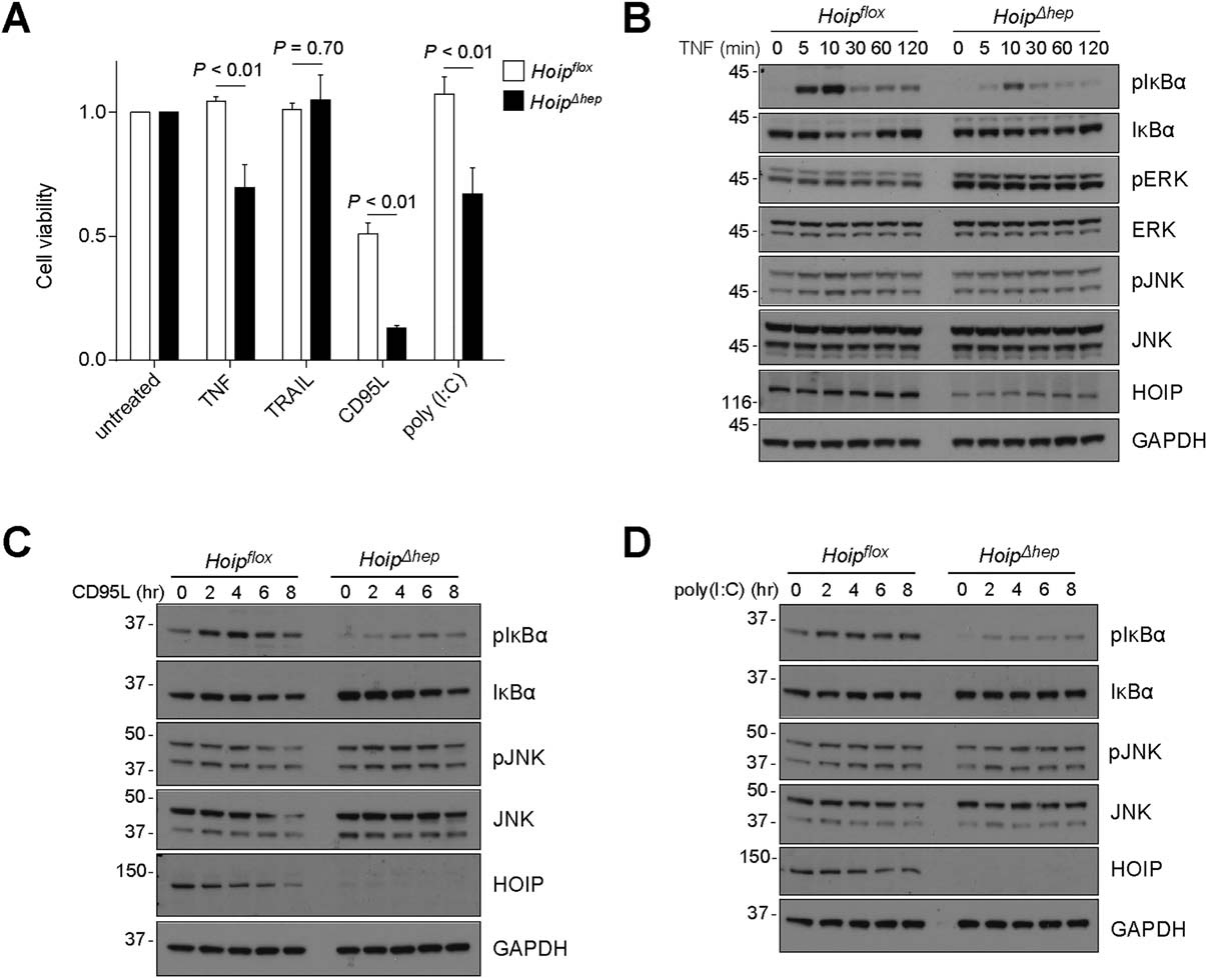
HOIP is required for suppression of cell death and optimal NF‐κB activation in hepatocytes in response to various immune receptor stimuli. (A) Cell viability assay of primary hepatocytes from *Hoip^flox^* and *Hoip^Δhep^* mice with the indicated treatments. Cells were treated with 1 μg/mL of TRAIL, CD95L‐Fc, and poly(I:C) for 24 hours (n = 3 each). Unpaired two‐tailed *t* test was employed for statistical analysis. (B‐D) Primary hepatocytes were stimulated with 10 ng/mL TNF (B), 1 μg/mL poly(I:C) (C), or 0.5 μg/mL CD95L‐Fc (D) for the indicated times; and hepatocyte lysates were immunoblotted for the indicated proteins. Abbreviations: GAPDH, glyceraldehyde 3‐phosphate dehydrogenase; pERK, phosphorylated ERK; pJNK, phosphorylated JNK.

It has been established that LUBAC deficiency leads to aberrant TNFR1 and other immune receptor signaling output,^17^, ^19^, ^20^, ^21^, ^23^ which could account for the increased liver cell death in *Hoip^Δhep^* mice. Indeed, HOIP‐deficient primary hepatocytes were more sensitive to cell death induction by TNF, CD95 ligand (CD95L/FasL), and polyinosinic:polycytidylic acid (poly(I:C)) but not TNF‐related apoptosis‐inducing ligand (TRAIL) (Fig. 4A). We also assessed whether HOIP‐deficient hepatocytes have a defect in gene activation. HOIP‐deficient primary hepatocytes showed substantially reduced phosphorylation and subsequent degradation of IκBα upon TNF stimulation and a slight reduction in JNK activation (Fig. 4B). Extracellular signal–regulated kinase (ERK) activation was not observed upon TNF stimulation, yet HOIP‐deficient cells had a higher basal level of phosphorylated extracellular signal–regulated kinase (Fig. 4B). In addition, phosphorylation of IκBα upon stimulation by CD95L or poly(I:C) was decreased in HOIP‐deficient hepatocytes, but phosphorylation of JNK was unaltered (Fig. 4C,D). These results illustrate that HOIP deficiency mainly dampens NF‐κB signaling in hepatocytes induced by multiple immune receptor stimuli. Notably, the basal level of phosphorylated IκBα was also reduced in HOIP‐deficient cells (Fig. 4B‐D, first lanes of *Hoip^flox^* and *Hoip^Δhep^* cells), implying that HOIP contributes to the induction of physiological levels of NF‐κB activation in hepatocytes in response to various stimuli, as reported for other cell types.^19^, ^20^, ^25^, ^31^

### HOIP SUPPRESSES FORMATION OF THE CELL DEATH–TRIGGERING SIGNALING COMPLEX INDEPENDENTLY OF ITS GENE‐ACTIVATING FUNCTION

**Figure 5:**
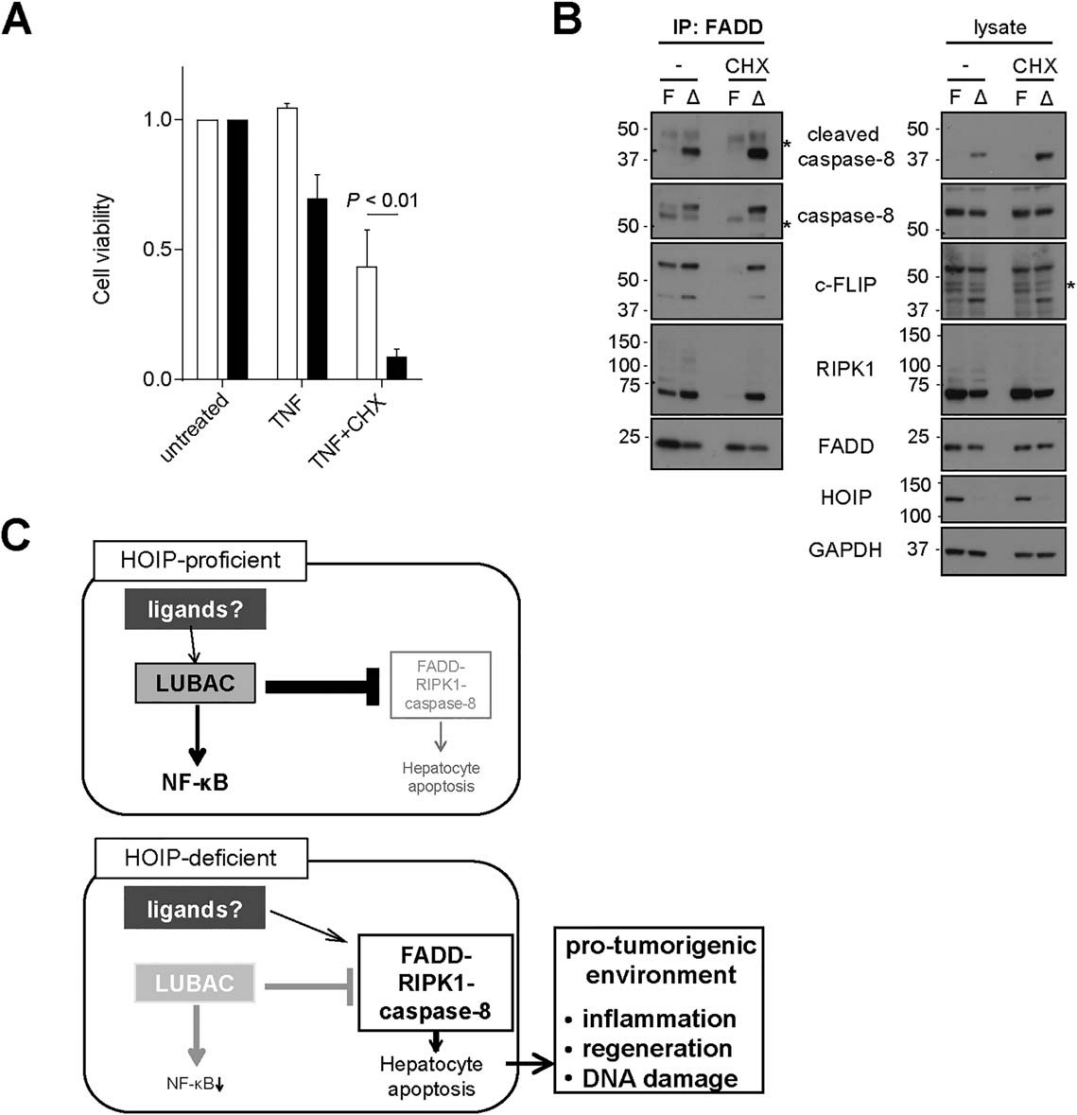
HOIP restrains formation of the cell death–inducing FADD–RIPK1–caspase‐8–c‐FLIP_L_–containing complex in hepatocytes, independently of its gene‐activating function. (A) Cell viability assay of primary hepatocytes isolated from *Hoip^flox^* and *Hoip^Δhep^* mice, treated with 1 μg/mL TNF for 24 hours in the presence or absence of 1 μg/mL CHX. Unpaired two‐tailed *t* test was employed for statistical analysis. (B) Immunoprecipitation of the FADD‐associated complex in z‐VAD‐fmk(zVAD)–treated (24 hours) primary hepatocytes from *Hoip^flox^* (F) and *Hoip^Δhep^* (Δ) mice in the absence or presence of 1 μg/mL CHX. Asterisks indicate unspecific bands. (C) Schematic representation of the role of LUBAC in HOIP‐proficient and HOIP‐deficient hepatocytes. Abbreviations: GAPDH, glyceraldehyde 3‐phosphate dehydrogenase; IP, immunoprecipitation.

Because impaired gene activation can result in increased susceptibility to cell death, we next evaluated whether or not the increased sensitivity of HOIP‐deficient hepatocytes to TNF‐induced cell death is a consequence of impaired NF‐κB–induced gene activation. To do so, we treated HOIP‐proficient and HOIP‐deficient cells with TNF in combination with cycloheximide (CHX). *Hoip^Δhep^* hepatocytes were still more susceptible to TNF/CHX‐induced death than control cells (Fig. 5A), demonstrating that HOIP can protect hepatocytes from TNF‐induced cell death independently of its role in gene activation. We previously showed that stimulation of HOIP‐deficient mouse embryonic fibroblasts results in aberrant formation of TNFR1 complex II, which contains FADD–caspase‐8–RIPK1 and is responsible for the increased death observed in these cells.^20^ Strikingly, HOIP‐deficient hepatocytes displayed robust formation of such a death‐triggering complex containing FADD, caspase‐8 (full‐length and cleaved), RIPK1, and cellular FLICE‐like inhibitory protein long isoform (c‐FLIP_L_), while its formation was limited in HOIP‐proficient primary hepatocytes (Fig. 5B, compare the first and second lanes). Intriguingly, this complex was readily formed in the absence of any exogenous stimulation. Hence, this evidence implies that HOIP‐deficient hepatocytes die as a consequence of the aberrant formation of this complex and caspase‐8 activation.

In sharp contrast to the marked increase in the formation of this complex by HOIP deficiency, abrogation of gene translation by treatment with the inhibitor CHX did not elevate formation of this complex in HOIP‐proficient hepatocytes (Fig. 5B, compare the first and fourth lanes). This result demonstrates that failure of gene activation, including that of NF‐κB target genes, does not account for the aberrant assembly of the FADD‐associated signaling complex that occurs in the absence of HOIP. These results imply that LUBAC exerts a suppressive function in hepatocytes toward formation of the FADD–RIPK1–caspase‐8–c‐FLIP_L_–containing complex and caspase‐8 activation independently of its gene‐activating capacity, which results in promoting protumorigenic events ensuing from cell death (Fig. 5C).

### TNFR1 DELETION IS NOT SUFFICIENT TO PREVENT THE DEATH OF HOIP‐DEFICIENT HEPATOCYTES

**Figure 6:**
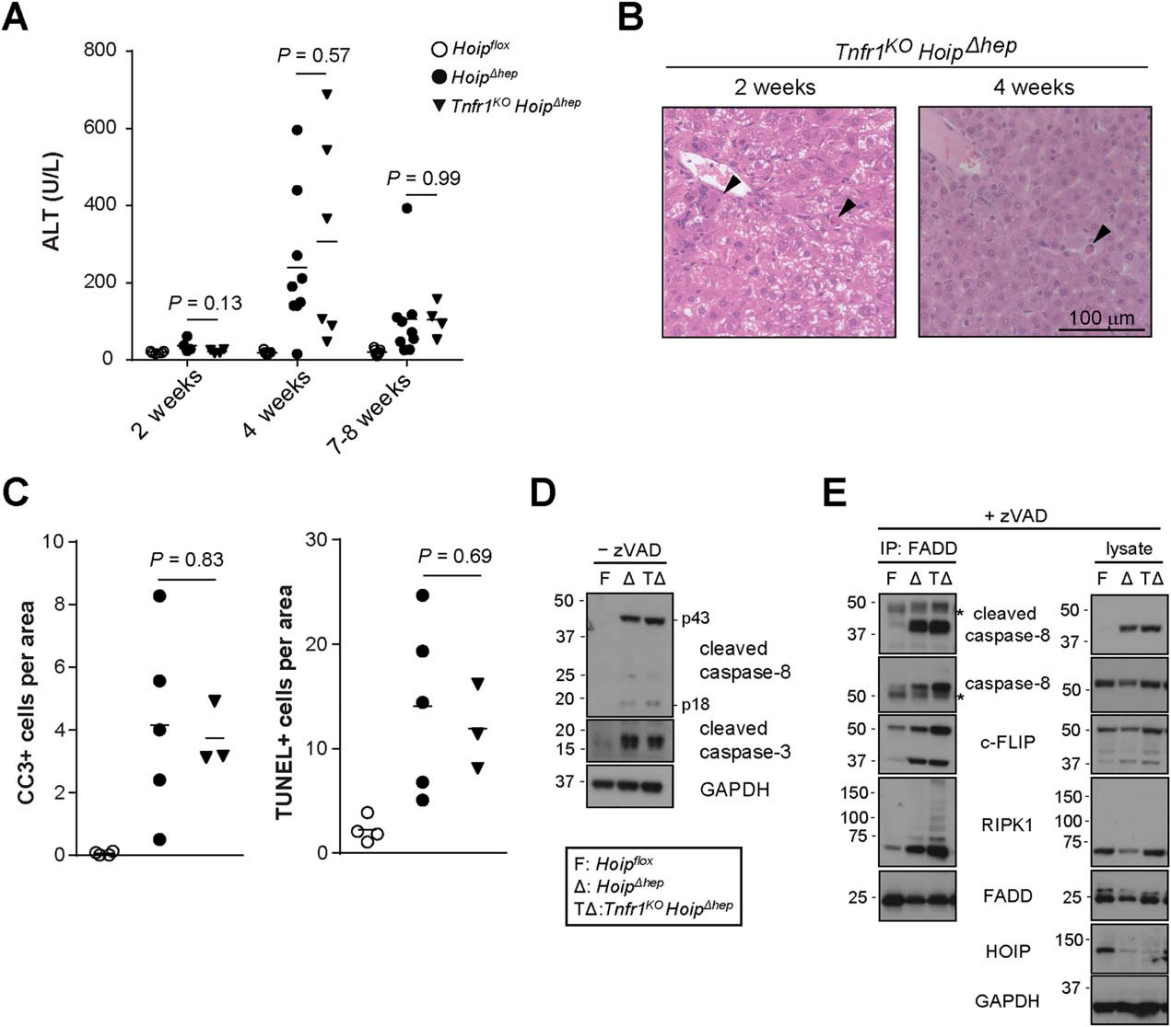
Hepatocyte apoptosis in HOIP‐deficient livers is unaffected by genetic ablation of TNFR1. (A) Levels of serum ALT in *Hoip^flox^*, *Hoip^Δhep^*, and *Tnfr1^KO^ Hoip^Δhep^* mice at the indicated ages. (B) Representative pictures of hematoxylin and eosin sections of the livers of *Tnfr1^KO^ Hoip^Δhep^* mice at 2 and 4 weeks of age. Arrowheads indicate apoptotic bodies. (C) Quantification of CC3^+^ and TUNEL^+^ cells per area of the livers of *Hoip^flox^*, *Hoip^Δhep^*, and *Tnfr1^KO^ Hoip^Δhep^* mice at 2 weeks of age. (D) Lysates of primary hepatocytes from *Hoip^flox^* (F), *Hoip^Δhep^* (Δ), and *Tnfr1^KO^ Hoip^Δhep^* (TΔ) mice at 8 weeks of age were immunoblotted for CC8 and CC3. (E) Immunoprecipitation of the FADD‐associated complex in zVAD‐treated (24 hours) primary hepatocytes from *Hoip^flox^* (F), *Hoip^Δhep^* (Δ), and *Tnfr1^KO^ Hoip^Δhep^* (TΔ) mice. Asterisks indicate unspecific bands. Unpaired two‐tailed *t* test was employed for statistical analysis. Abbreviations: GAPDH, glyceraldehyde 3‐phosphate dehydrogenase; IP, immunoprecipitation.

HOIP‐deficient hepatocytes are more sensitive to TNF‐induced cell death (Fig. 4A). In addition, previous work has demonstrated that the aberrant cell death observed in SHARPIN‐deficient and HOIP‐deficient mice is rescued by genetic ablation of TNFR1.^19^, ^20^, ^21^, ^23^ We therefore hypothesized that exacerbated cell death and inflammation in HOIP‐deficient livers was also mediated by TNFR1 signaling. Unexpectedly, however, when we crossed *Hoip^Δhep^* mice with mice constitutively devoid of TNFR1 (*Tnfr1^KO^*) mice, the damage in the resulting *Tnfr1^KO^ Hoip^Δhep^* livers was similar to that in *Hoip^Δhep^* livers as assessed by serum ALT values and histological analysis (Fig. 6A,B). Furthermore, codeletion of TNFR1 did not reduce the numbers of TUNEL^+^ and CC3^+^ cells in HOIP‐deficient livers (Fig. 6C). In line with these results, activation of caspase‐8 and caspase‐3 was equivalent in *Tnfr1^KO^ Hoip^Δhep^* and *Hoip^Δhep^* hepatocytes (Fig. 6D) and TNFR1 ablation did not prevent formation of the FADD–RIPK1–caspase‐8–c‐FLIP_L_–containing complex in HOIP‐deficient hepatocytes (Fig. 6E).

As HOIP‐deficient hepatocytes were highly sensitive to CD95L (Fig. 4A), we next sought to determine whether stimulation of CD95 could be responsible for increased cell death in *Tnfr1^KO^ Hoip^Δhep^* livers.We, therefore, crossed *Tnfr1^KO^ Hoip^Δhep^* mice with *Cd95‐DD^flox^* mice to delete the DD of CD95 in LPCs. We confirmed that hepatocytes from *Cd95‐DD^Δhep^ Tnfr1^KO^ Hoip^Δhep^* mice failed to activate caspases upon CD95L stimulation (Supporting Fig. S5A). Despite the functionally complete deletion of both TNFR1 and the DD of CD95, *Cd95‐DD^Δhep^Tnfr1^KO^ Hoip^Δhep^* mice had significantly higher ALT in the serum (Supporting Fig. S5B). In accordance, hepatocytes isolated from these animals contain the FADD‐associated caspase‐8–activating complex to a similar extent as those obtained from *Hoip^Δhep^* mice (Supporting Fig. S5C). Thus, ablating the capacity of CD95 to induce cell death, in addition to fully ablating TNFR1, does not prevent the increase in aberrant cell death which characterizes HOIP‐deficient livers.

### TNFR1 MEDIATES HEPATITIS AND TUMORIGENESIS UPON LOSS OF HOIP IN THE LIVER

**Figure 7:**
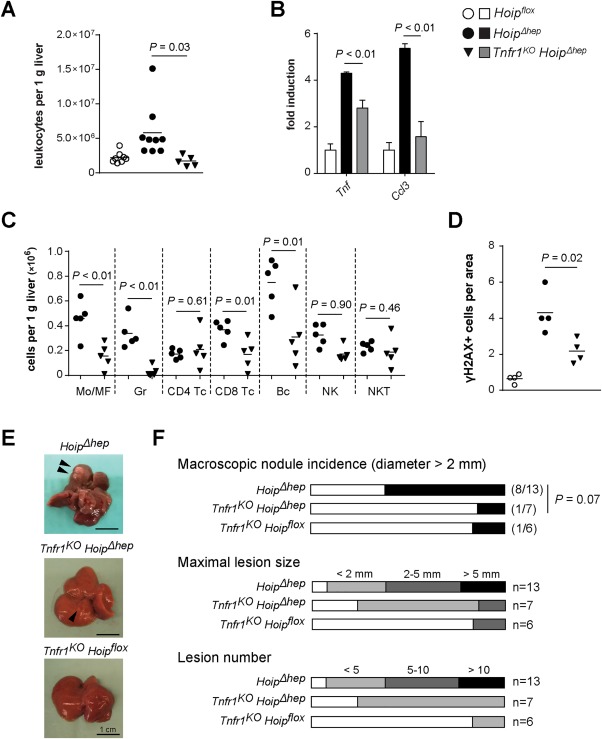
TNFR1 mediates inflammation and promotes carcinogenesis in HOIP‐deficient livers. (A) Count of hepatic CD45^+^ cells per gram of liver in *Hoip^flox^*, *Hoip^Δhep^*, and *Tnfr1^KO^ Hoip^Δhep^* mice at 4 weeks of age. (B) Relative quantification of the transcript levels of *Tnf* and *Ccl3* in the liver of *Hoip^flox^*, *Hoip^Δhep^*, and *Tnfr1^KO^ Hoip^Δhep^* mice at 4 weeks of age. Levels of transcripts were normalized to those of *Hoip^flox^* livers. (C) Liver cells from *Hoip^Δhep^* and *Tnfr1^KO^ Hoip^Δhep^* mice at 4 weeks were stained, and the numbers of hepatic immune subpopulations were quantified by flow cytometry. (D) Quantification of γH2AX^+^ cells per field of the liver of *Hoip^flox^*, *Hoip^Δhep^*, and *Tnfr1^KO^ Hoip^Δhep^* mice at 4 weeks of age. Unpaired two‐tailed *t* test was employed for statistical analysis (A‐D). (E) Representative pictures of livers of *Hoip^Δhep^*, *Tnfr1^KO^ Hoip^Δhep^*, and *Tnfr1^KO^ Hoip^flox^* mice at 18 months of age (left panels). (F) Comparison between *Hoip^Δhep^*, *Tnfr1^KO^ Hoip^Δhep^*, and *Tnfr1^KO^ Hoip^flox^* mice on the incidence of macroscopic nodules (diameter >2 mm) (upper graph), maximal lesion size (middle graph), and lesion number (lower graph). Abbreviations: Bc, B220^+^ cells; CD4 Tc, CD3^+^ CD4^+^ cells; CD8 Tc, CD3^+^ CD8^+^ cells; Gr, Ly6G^+^ cells; Mo/MF, F4/80^+^ cells; NK, CD3^−^ NK1.1^+^ natural killer cells; NKT, CD3^+^ NK1.1^+^ natural killer T cells.

We next evaluated the effect of TNFR1 ablation on hepatitis in *Hoip^Δhep^* mice. Remarkably, even though cell death was unaffected in *Tnfr1^KO^ Hoip^Δhep^* livers (Fig. 6), the number of leukocytes present in these livers was significantly lower than in *Hoip^Δhep^* livers (Fig. 7A). The transcript levels of *Tnf* and *Ccl3* were also markedly decreased in *Tnfr1^KO^ Hoip^Δhep^* livers (Fig. 7B). Furthermore, the numbers of hepatic myeloid cells as well as CD8^+^ T cells and B cells were diminished by TNFR1 ablation (Fig. 7C). In accordance with attenuated inflammation, DNA damage in *Hoip^Δhep^* livers was significantly alleviated by deletion of TNFR1, as confirmed by γH2AX staining (Fig. 7D). To evaluate the effect of TNFR1 deletion in tumorigenesis driven by HOIP deficiency, we also analyzed livers of *Tnfr1^KO^ Hoip^Δhep^* mice at 18 months of age. The incidence of macroscopic nodules was considerably reduced in these mice compared to *Hoip^Δhep^* mice. Moreover, *Tnfr1^KO^ Hoip^Δhep^* mice displayed decreased lesion numbers and the lesions that did arise were considerably smaller in size than in *Hoip^Δhep^* mice (Fig. 7E,F). Collectively, these results demonstrate that TNFR1 signaling contributes to HOIP deficiency–mediated liver inflammation and carcinogenesis even though exacerbated hepatocyte death, likely the event that triggers these consequences, still occurs in the absence of TNFR1.

## Discussion

Here, we evaluated the impact of LUBAC deficiency on liver homeostasis by genetically ablating HOIP, the catalytically active component of LUBAC, in mouse LPCs. LUBAC deficiency in LPCs results in hepatocyte apoptosis, followed by inflammation in the liver within the first month of life and tumorigenesis at 18 months of age. Intriguingly, cell death regulation by LUBAC in hepatocytes is independent of its function in gene activation and, importantly, requires neither TNFR1 nor the DD of CD95 *in vivo*. Yet, TNFR1 contributes to the inflammatory processes that are likely triggered by the aberrant death of HOIP‐deficient hepatocytes and which eventually results in liver carcinogenesis.

The first aberration we detected in HOIP‐deleted livers was an increased level of apoptosis at postnatal day 7 before inflammation became noticeable. Patients suffering from chronic hepatitis, including viral hepatitis and nonalcoholic steatohepatitis, display hepatocyte death.^32^ In addition, apoptosis can be a potent driver of inflammation and liver tumorigenesis.^11^, ^33^ Apoptotic cells elicit activation of the immune system and production of mediators of inflammation, which can also activate the regenerative process of hepatocytes. In fact, cytokines and growth factors involved in tissue regeneration are linked to tumor promotion.^34^, ^35^ Furthermore, it is possible that the DNA‐damaging environment in HOIP‐deficient livers is promoted by impaired NF‐κB activation in addition to cell death, which was illustrated in the studies using LPC‐specific IKKβ knockout mice.^8^, ^34^ We therefore postulate that HOIP deletion initially affects liver homeostasis by promoting hepatocyte apoptosis and that this, through facilitating liver inflammation, regeneration, and DNA damage, creates a protumorigenic environment which enables the formation of HCC.

*Alb‐Cre*–mediated target gene deletion occurs gradually from late gestation to approximately 6 weeks of age.^24^ Because HOIP deletion in hepatocytes of *Hoip^Δhep^* mice is efficiently executed at 8‐10 weeks of age, HOIP deletion should be completed by this stage and no new HOIP‐depleted cell are generated. Loss of HOIP causes hepatocyte death, yet a fraction of HOIP‐deficient cells appear to be able to survive. As the number of HOIP‐deleted cells approaches a plateau, the number of newly HOIP‐deleted cells declines, which would provide a decreased source of apoptotic cells, hence explaining the kinetics of cell death observed in *Hoip^Δhep^* mice. Remarkably, even though cell death and inflammation are resolved during adulthood in *Hoip^Δhep^* mice, the events occurring at early stages of life are sufficient to cause liver tumorigenesis late in life.

Unexpectedly, unlike our previous observations in SHARPIN‐deficient *cpdm* mice^19^, ^21^, ^23^ and HOIP‐deficient embryos,^20^ LUBAC deficiency–mediated hepatocyte apoptosis does not require TNFR1. Yet intriguingly, the subsequent enhanced leukocyte recruitment observed in mice proficient for TNFR1 is reverted to normal levels by systemic ablation of TNFR1 in mice with HOIP‐deficient livers. This evidence indicates that, while the cell death does not require TNFR1, this receptor contributes to the ensuing inflammation. Thus, TNFR1 signaling from nonparenchymal liver cells, including from immune, hepatic stellate, and endothelial cells, might be involved in the development of hepatitis in mice with HOIP‐deficient livers. Furthermore, in line with decreased inflammation, TNFR1 ablation significantly reduced γH2AX^+^ cells in HOIP‐deficient livers. Apart from inflammation, caspase activation observed in HOIP‐deficient hepatocytes could also be responsible for the induction of DNA damage by resulting in DNA fragmentation. In fact, DNA damage was not completely inhibited despite the apparent absence of inflammation by TNFR1 deficiency. These results suggest that DNA damage in the liver may be induced as a consequence of both TNFR1‐independent caspase activation and TNFR1‐dependent inflammation. Previous studies revealed the tumor‐promoting role of TNF in carcinogen‐induced liver cancer.^35^ In accord, TNFR1 ablation prevented tumorigenesis triggered by HOIP deficiency, presumably by inhibiting inflammation and subsequent DNA damage.

HOIP‐deficient hepatocytes are sensitized to death induction by triggering of CD95 and toll‐like receptor 3 but not TRAIL. While this result suggests CD95 and toll‐like receptor 3 as possible candidate receptors for mediating hepatocyte death in *Hoip^Δhep^* mice, the liver cell death observed in *Hoip^Δhep^* mice could be mediated by entirely other systems capable of inducing cell death and for which a role of LUBAC has not yet been described. In fact, in addition to CD95L and poly(I:C), TNF was capable of enhanced killing of HOIP‐deficient compared to HOIP‐proficient hepatocytes, yet ablation of TNFR1 alone or the combined deletion of TNFR1 and the DD of CD95 was not sufficient to prevent cell death in the livers of these mice. Of note, inflammation driven by NF‐κB essential modulator deficiency in the liver parenchyma is not ameliorated by concomitant LPC‐specific abrogation of TNFR1, TRAIL receptor, and/or the DD of CD95.^36^ It is therefore tempting to speculate that a yet unidentified immune receptor, or indeed a combination of multiple immune receptors, might be responsible for hepatocyte cell death and the ensuing inflammation upon HOIP deficiency *in vivo*.

Alternatively, aberrant cell death could be a consequence of spontaneous formation of the killing complex in the absence of an exogenous stimulus. Indeed, loss of cellular inhibitor of apoptosis proteins by Smac mimetic treatment alone results in spontaneous formation of a FADD–RIPK1–caspase‐8–containing complex, termed the “ripoptosome,”^37^, ^38^ which is reminiscent of the signaling complex present in HOIP‐deficient hepatocytes. The ripoptosome is assembled independently of autocrine TNF, TRAIL, and CD95L.^37^, ^38^ Additional recent studies illustrated the crucial role of RIPK1 and its kinase activity in the formation of the FADD–RIPK1–caspase‐8 apoptotic signaling complex.^13^, ^39^, ^40^ In particular, aberrant cell death causative for the inflammatory phenotype that characterizes *cpdm* mice is completely prevented by genetic inactivation of RIPK1's kinase activity.^41^ It would therefore be interesting to interrogate the role of the kinase activity of RIPK1 in aberrant death complex formation and apoptosis driven by HOIP deficiency in hepatocytes.

HOIP deletion in the liver parenchyma results in cancer formation with overexpression of mitotic genes. A class of human HCC with robust up‐regulation of mitosis and cell cycle regulators was identified by Thorgeirsson and colleagues, who termed this subclass A and reported a strong correlation with poor prognosis of patients with HCC.^28^ Moreover, according to the classification of human HCC by Boyault et al. into subgroups G1‐G6, this signature resembles that of the G3 subgroup of hepatitis B virus–free HCC with highly overexpressed cell cycle regulatory genes.^27^ In addition, these nodules displayed up‐regulation of fetal liver genes as observed in the G1 subgroup. Both the G1 and G3 subgroups belong to the meta‐group G1‐G3, which is linked to higher genomic instability and which corresponds to subclass A. In summary, the tumor nodules found in *Hoip^Δhep^* mice appear to recapitulate the profile of hepatitis B virus–negative human HCC with chromosomal instability and a more unfavorable outcome.

The pathology of *Hoip^Δhep^* livers does not mirror the currently most common process of liver carcinogenesis, arising as a consequence of cirrhosis. However, there is an increasing trend of patients suffering from HCC, particularly in the Western world, associated with obesity and metabolic syndrome but without prior history of cirrhosis.^42^ In our mouse model, tumor‐bearing HOIP‐deficient livers exhibited steatotic features, which may indicate some metabolic alterations in hepatocytes. Therefore, *Hoip^Δhep^* livers could imitate the progression of nonalcoholic fatty liver disease to HCC, bypassing a cirrhotic condition. It would therefore be tempting to speculate that HOIP deficiency in the liver could mimic the conditions in nonalcoholic fatty liver disease patients and could possibly serve as a model for its etiology. The pathologies found in *Hoip^Δhep^* livers appear to be driven by aberrant cell death, which is often seen in hepatitis patients. It is therefore possible to envisage that blockade of cell death may be of therapeutic benefit for patients suffering liver complications including nonalcoholic fatty liver disease. Indeed, the caspase inhibitor emricasan (IDN‐6556) has been shown to alleviate liver damage in human patients,^43^ and a clinical trial testing its effects on nonalcoholic steatohepatitis and liver fibrosis is currently under way (ClinicalTrials.gov identifier NCT02686762).

In conclusion, LUBAC is a previously unidentified tumor suppressor inhibiting hepatocyte apoptosis at postnatal stages that does not require TNFR1. In the absence of LUBAC, a tumor‐conducive inflammatory environment emerges in these livers which have suffered from hepatocyte apoptosis followed by compensatory proliferation. The development of this environment involves hepatitis and DNA damage, which, in turn, are dependent on TNFR1. These results identify LUBAC as a crucial factor ensuring hepatocyte survival and as a suppressor of hepatitis and hepatocarcinogenesis.

Author names in bold designate shared co‐first authorship.

## Supporting information

Additional Supporting Information may be found at onlinelibrary.wiley.com/doi/10.1002/hep.29074/suppinfo.

Supporting Information
