# Supplementary material for: The Linear ubiquitin chain assembly complex acts as a liver tumor suppressor and inhibits hepatocyte apoptosis and hepatitis
Source: Hepatology. 2017 Apr 10;65(6):1963–78. doi: 10.1002/hep.29074 (PMC5485060; doi:10.1002/hep.29074)
Supplement: Supplementary file 1 — Supporting Information [file HEP-65-1963-s001.pdf]

Supporting information

HISTOLOGY AND IMMUNOHISTOCHEMISTRY

Resected livers were fixed in 10% formalin (Sigma-Aldrich, St. Louis, MO) and embedded in paraffin blocks and cut into sections of 4 μm in thickness. H&E staining was performed to observe tissue structure. Sirius Red staining was performed to detect fibrotic lesions. As for antigen retrieval, heat-induced epitope retrieval with citrate buffer (10 mM sodium citrate, pH 6.0) was used for Ki-67, CK19 and GS staining. For Ly6G and γH2AX staining, Tris-EDTA buffer (Tris 10 mM, EDTA 1 mM, pH 8.0) was employed. For F4/80 staining, proteinase K (10 μg/mL) diluted in TEX buffer (50 mM Tris, 1 mM EDTA, 0.5% Triton X-100, pH 8.0) was utilized. Used primary antibodies are listed in Supporting Table. HRP-conjugated antibodies (Southern Biotech, Birmingham, AL) were used as secondary antibodies. For Ki-67, Ly6G and γH2AX staining, tyramide signal amplification system (Perkin Elmer, Waltham, MA) was utilized to enhance the signals. 3,3'-diaminobenzidine (DAB) was used as a substrate for CK19 and GS staining. To detect cell death, cleaved caspase-3 staining was performed with epitope retrieval using citrate buffer followed by terminal deoxynucleotidyl transferase dUTP nick end labeling (TUNEL) using ApopTag Red In Situ Apoptosis Detection Kit (Merck Millipore, Billerica, MA). Nuclear counterstain was performed with DAPI, which is indicated in blue in the figures.

IMMUNOPRECIPITATION

For an immunoprecipitation for a FADD-associated complex, hepatocyte lysates from $\textit{Hoip}^{flox}$ and $\textit{Hoip}^{\Delta hep}$ mice were pre-cleared with Sepharose CL-4B (Sigma-Aldrich) for 2 hours. Protein G-coupled sepharose (GE Healthcare, Little Chalfont, UK) was blocked with 1% BSA in IP lysis buffer for 1 hour and washed extensively with IP lysis buffer prior to incubation. Pre-cleared lysates were incubated overnight with blocked Protein G-coupled sepharose and anti-FADD (M-19) antibody (Santa Cruz, Dallas, TX) at 4ºC. Beads were washed 5 times with ice-cold IP lysis buffer and vortexed, and proteins were eluted in 1x LDS buffer with 12.5 mM DTT at 92ºC. TNF-RSC pull

down with anti-FLAG M2 beads were previously described by Haas et al., 2009 and Draber et al., 2015.

FLOW CYTOMETRIC ANALYSIS OF LIVER IMMUNE CELLS

Livers were mechanically dissociated in PBS with a plunger of 5 mL syringes and cell suspensions were filtered through nylon mesh. Cells were washed with PBS and red blood cells were removed with RBC lysis buffer (R&D systems, Minneapolis, MN). Cells were subsequently incubated with anti-mouse CD16/CD32 (eBioscience, San Diego, CA) and Fixable Viability Dye eFluor 660 (eBioscience) for 30 min prior to incubation with antibodies. Cells were stained with fluorophore-coupled antibodies (listed in Supporting Table) for 1 hour. For absolute quantification, 123count eBeads (eBioscience) was added to cell suspensions and cells were measured on Fortessa LSR II (BD Biosciences, Franklin Lakes, NJ). Analysis was performed using FlowJo 7.6.5 (Tree Star, Ashland, OR).

ISOLATION OF MOUSE PRIMARY HEPATOCYTES

Primary hepatocytes were isolated from seven-to-twelve week-old mice. Mice were anesthetized and were cannulated via inferior vena cava and portal vein was then incised. Mouse livers were perfused with warm perfusion buffer (NaCl 8 g/L, KCl 0.2 g/L, Na_2HPO_4 33 mg/L, HEPES 2.38 g/L, pH adjusted to 7.65 with NaOH) supplemented with 0.5 mM EDTA for 7 minutes. The second perfusion with warm perfusion buffer plus 0.5 mg/mL collagenase from *Clostridium histolyticum* (Sigma-Aldrich) and 10 mM $CaCl_2$ was subsequently performed for 3 minutes. Digested livers were isolated in warm hepatocyte culture medium (William's E medium Glutamax supplement (Thermo Fisher Scientific, Waltham, MA) with 1x Antibiotic-antimycotic (Thermo Fisher Scientific), 0.1 % BSA (Life technologies, Carlsbad, CA) and 25 nM dexamethasone (Sigma-Aldrich)) with 10% FCS. Cell pellets were washed with hepatocyte culture medium twice with FCS (Sigma-Aldrich). Isolated hepatocytes were plated on plates coated with collagen type I (Sigma-Aldrich) in hepatocyte culture medium with 10% FCS. The culture media were replaced 3-4 hours after plating with hepatocyte culture medium without FCS.

CELL VIABILITY ASSAY

Culture media were removed and primary hepatocytes were lysed in Cell Titer Glo solution (Promega, Madison, WI) diluted 1:6 in PBS. Cells were incubated on a shaker for 10 minutes, and lysates were transferred to opaque 96-well plates for measurement. Luminescence was quantified with Mithras (Berthold, Bad Wildbad, Germany).

TISSUE RNA EXTRACTION AND RT-QPCR

Snap-frozen liver pieces were grinded into fine powder with a mortar and a pestle and RNA was extracted from livers using RNeasy Mini kit (Qiagen, Hilden, Germany). Purified RNA was subsequently employed for cDNA synthesis using RevertAid First Strand cDNA Synthesis Kit and poly-A primer (Thermo Scientific). Synthesised cDNA was used for RT-PCR, with FastStart Universal Probe Master (Rox) and Universal Probe system (Roche, Basel, Switzerland) and specific sets of primers:

Tnf forward	CTGTAGCCCACGTCGTAGC
Tnf reverse	TTGAGATCCATGCCGTTG
Hprt forward	TGATAGATCCATTCCTATGACTGTAGA
Hprt reverse	AAGACATTCTTTCCAGTTAAAGTTGAG
Ccl3 forward	TGCCCTTGCTGTTCTTCTCT
Ccl3 reverse	GTGGAATCTTCCGGCTGTAG
Cxcl1 forward	GACTCCAGCCACACTCCAAC
Cxcl1 reverse	TGACAGCGCAGCTCATTG

WESTERN BLOTTING

Proteins separated on polyacrylamide gels were transferred to nitrocellulose membranes by wet or semi-dry transfer. Membranes were blocked with 2.5 % skimmed milk (Sigma-Aldrich) in PBS-T plus 0.02% sodium azide (Sigma-Aldrich) for 1 hour, and were washed briefly with PBS-T and were incubated with primary antibodies (listed in Supplementary Table) diluted in 2.5% BSA (VWR) in PBS-T plus 0.1% sodium azide. Membranes were washed briefly with PBS-T and were subsequently

incubated with host/isotype-matched HRP-conjugated secondary antibodies (Southern Biotech). Membranes were developed with ECL plus substrate (Thermo Fisher Scientific).

RNA-SEQ

Total RNA was extracted from non-tumor and nodular liver tissues from eighteen month-old *Hoip*$^{\Delta hep}$ mice using RNeasy mini (Qiagen). For library preparation, samples were processed using Kapa Stranded RNA-Seq with RiboErase sample preparation kit (Kapa Biosystems, Wilmington, MA) according to manufacturer's instructions. Briefly, single-stranded DNA probes complementary to mouse ribosomal RNA (rRNA) sequence were hybridized to 500 ng of each total RNA sample. RNase H was then used to degrade DNA-RNA hybrids while leaving any single stranded RNA intact. DNA probes were subsequently degraded using DNase I. The remaining RNA was then purified, fragmented using chemical fragmentation (heat and divalent metal cation) and primed with random hexamers. Strand-specific first strand cDNA was generated using reverse transcriptase in the presence of actinomycin D. This allows for RNA-dependent synthesis while preventing spurious DNA-dependent synthesis. The second cDNA strand was synthesized using dUTP in place of dTTP, to mark the second strand. The resultant cDNA is then A-tailed at the 3' end to prevent self-ligation and adapter dimerisation. Truncated adaptors, containing a T-overhang are ligated to the A-tailed cDNA. Successfully ligated cDNA molecules were then enriched with limited cycle PCR (10 cycles). Of note, the first strand is amplified for sequencing. The primers used extend the adaptor to full length and contain sequences that allow each libraries to be uniquely identified by way of a 6bp index sequence. Libraries are pooled in a single run in equimolar quantities, calculated from Qubit (Thermo Fisher Scientific) and bioanalyser fragment analysis. Samples were then sequenced on NextSeq 500 instrument (Illumina, San Diego, CA) using a 43bp paired end protocol.

The sequence data in FASTQ sequence results were aligned by RNA-Seq alignment app (Illumina, Basescope) to obtain read counts of each gene. Spliced transcripts alignment to a reference (STAR) was employed as the preferred aligner. Read counts of each gene were then translated to a relative expression profiling analysis using DESeq2 (Illumina Basescope) to compare the expression profiles

of nodular samples versus non-tumor samples. 714 genes are identified to be statistically significantly upregulated or downregulated (q-value < 0.05) in the nodular samples and plotted to a heatmap by clustering analysis. Top 182 genes differentially expressed in the tumor samples ($\log_2$(tumor/non-tumor>1.2) were subjected to enriched neighborhood-based sets (NESTs) and enriched gene ontology(GO)-based sets analyses using the ConsensusPathDB platform (Max Planck Institute for Molecular Genetics, Germany, http://www.molgen.mpg.de/).

Supporting Table: primary antibody lists

Western blot

antibody	manufacturer	catalogue number	host/isotype
Bax	Santa Cruz	sc-7480	mouse IgG2b
Bcl-2	Santa Cruz	sc-7382	mouse IgG1
Bcl-X_L	Cell signaling	2764	rabbit
Bid	R&D systems	AF860	goat
caspase-8	Enzo Life Sciences	ALX-804-447	rat
caspase-9	Cell Signaling	9508	mouse IgG1
c-FLIP	Adipogen	AG-20B-0005	rat
cIAP1/2	R&D systems	315301	mouse IgG2a
cleaved caspase-3	Cell Signaling	9661	rabbit
cleaved caspase-8	Cell Signaling	9429	rabbit
ERK1/2	Cell Signaling	9102	rabbit
FADD	Assay Design	AAM-121	mouse IgG1
GAPDH	Abcam	ab8245	mouse IgG1
HOIL-1	homemade	-	mouse IgG2a
HOIP	homemade	-	rabbit
IκBα	Cell Signaling	9242	rabbit
JNK	Cell Signaling	9258	rabbit
Mcl-1	Cell signaling	94296	rabbit
phospho-ERK (T202/Y204)	Cell Signaling	9101	rabbit
phospho-IκBα (S32/S36)	Cell Signaling	9246	mouse IgG1
phospho-JNK (T183/Y185)	Cell Signaling	4671	rabbit
RIPK1	BD Biosciences	610459	mouse IgG2a
RIPK3	Enzo Life Sciences	ADI-905-242	rabbit
SHARPIN	Proteintech	14626-1-AP	rabbit
XIAP	Cell Signaling	2042	rabbit

Immunohistochemistry

antibody	manufacturer	catalogue number	host/isotype
CK19	BioRad	AHP1846	rabbit
F4/80	AbD serotec	MCA497G	rat
GS	Abcam	ab73593	rabbit
γH2AX	Cell Signaling	9718	rabbit
HNF4	Abcam	ab41898	mouse IgG2a
Ki-67	Abcam	ab16667	rabbit
Ly6G	Biolegend	4767	rat

Flow cytometry

antibody	manufacturer	catalogue number	fluorophore
B220	Biolegend	103243	BV650
CD3	Biolegend	100326	PerCP
CD4	eBioscience	25-0041-82	PE-Cy7
CD45	Biolegend	103128	Alexa Fluor 700
CD8	Biolegend	100714	APC-Cy7
F4/80	Biolegend	123110	PE
Ly6G	Biolegend	127606	FITC
NK1.1	Biolegend	108731	BV421

Supporting Figure Legends

Supporting Fig. S1.

(A) Levels of LUBAC components in primary hepatocytes from $Hoip^{flox}$ and $Hoip^{\Delta hep}$ mice at eight-to-nine weeks of age. (B) TNF-RSC pull-down from hepatocyte lysates of $Hoip^{flox}$ and $Hoip^{\Delta hep}$ mice and the precipitates were immunoblotted for linear ubiquitin and LUBAC components. Hepatocytes were stimulated with 200 μg/mL FLAG-tagged TNF for 15 minutes. (C) Survival curves for $Hoip^{flox}$ (open circle) and $Hoip^{\Delta hep}$ mice (closed circle) up to eighteen months ($Hoip^{flox}$: n=11, $Hoip^{\Delta hep}$: n=15).

Supporting Fig. S2.

(A) Representative macroscopic pictures of eighteen month-old livers of $Hoip^{flox}$ and $Hoip^{\Delta hep}$ mice. Black arrowheads indicate large nodules and white arrowheads indicate cystic lesions. (B) Cystic lesions in eighteen month-old livers from $Hoip^{\Delta hep}$ mice. (C) GS and CK19 staining of non-tumoral and tumoral areas of livers obtained from $Hoip^{\Delta hep}$ mice. (D) H&E staining of representative steatotic lesions in $Hoip^{\Delta hep}$ mice. S: steatotic lesions.

Supporting Fig. S3.

Immunostaining of F4/80 and Ly6G in the livers of *Hoip*^{flox} and *Hoip*$^{\Delta hep}$ mice at four weeks of age, indicated in red. White arrowheads show accumulation of stained cells.

Supporting Fig. S4.

(A) A plot of serum levels of ALT and alkaline phosphatase (AP) (left panel) and serum bilirubin levels (right panel) of *Hoip*^{flox} and *Hoip*$^{\Delta hep}$ mice at 4 weeks of age. (B) Representative picture of cleaved caspase-3 and TUNEL staining in the livers of *Hoip*^{flox} and *Hoip*$^{\Delta hep}$ mice at two weeks of age. (C) The liver lysates of *Hoip*^{flox} and *Hoip*$^{\Delta hep}$ mice at four weeks of age was immunoblotted for cleaved caspase-8. (D) Levels of pro-apoptotic and anti-apoptotic proteins evaluated in hepatocytes from *Hoip*^{flox} and *Hoip*$^{\Delta hep}$ mice. (E) Phosphorylation of JNK and IκBα was evaluated in hepatocytes from *Hoip*^{flox} and *Hoip*$^{\Delta hep}$ mice.

Supporting Fig. S5.

(A) Hepatocytes from *Cd95-DD*$^{\Delta hep}$ *Tnfr1*^{KO} *Hoip*$^{\Delta hep}$ mice (CΔ) were treated with CD95L-Fc as well as *Hoip*$^{\Delta hep}$ (Δ) cells and the lysates were immunoblotted for active caspases. (B) The levels of serum alanine aminotransferase (ALT) in *Cd95-DD*^{flox} *Tnfr1*^{KO} *Hoip*^{flox} (open square) and *Cd95-DD*$^{\Delta hep}$ *Tnfr1*^{KO} *Hoip*$^{\Delta hep}$ mice (closed square) at six weeks of age. (C) An immunoprecipitation of the FADD-associated complex in zVAD-treated (24 hours) primary hepatocytes from *Hoip*^{flox} (F), *Hoip*$^{\Delta hep}$ (Δ) and *Cd95-DD*$^{\Delta hep}$ *Tnfr1*^{KO} *Hoip*$^{\Delta hep}$ (CΔ) mice.

Fig S1

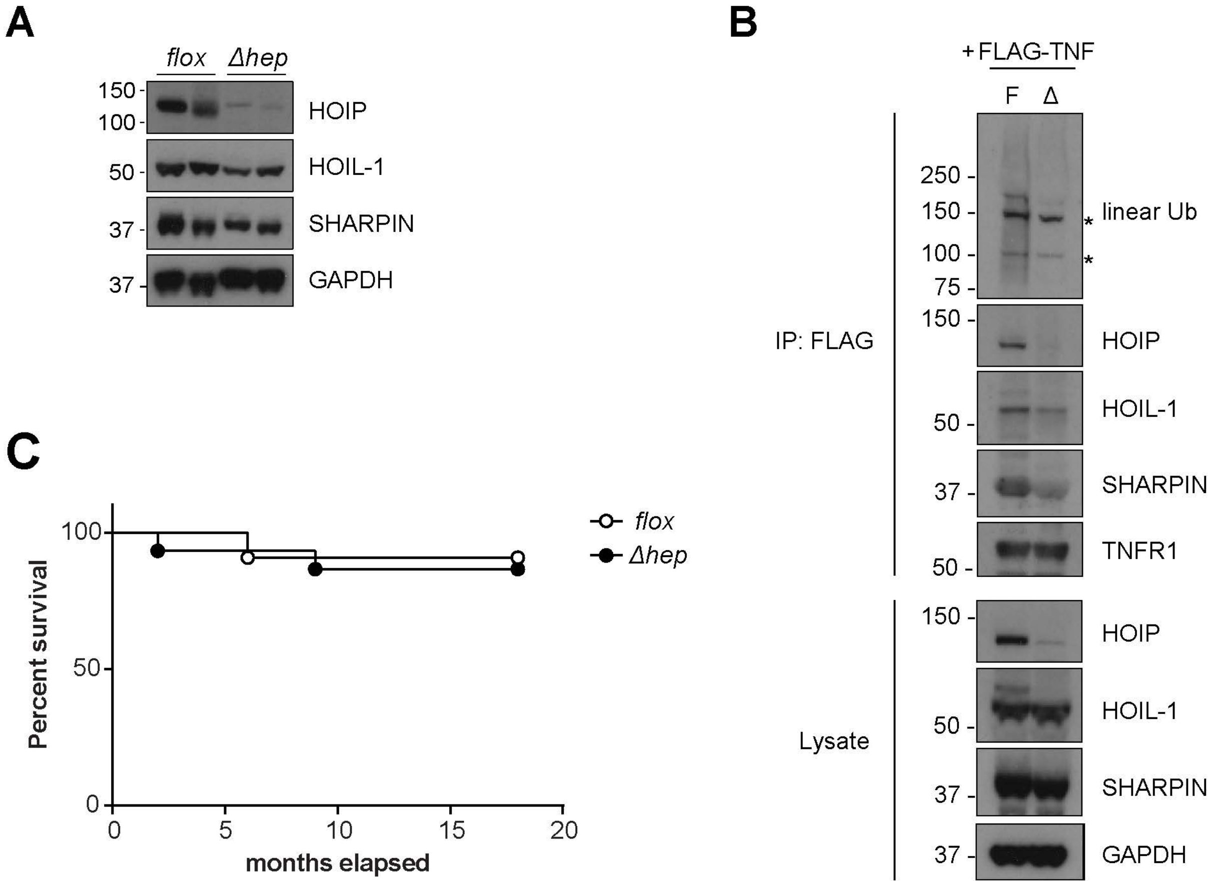
A
flox
Δhep
150
100
HOIP
50
HOIL-1
37
SHARPIN
37
GAPDH
B
+ FLAG-TNF
F
Δ
IP: FLAG
250
150
100
75
linear Ub
*
*
150
HOIP
50
HOIL-1
37
SHARPIN
50
TNFR1
Lysate
150
HOIP
50
HOIL-1
37
SHARPIN
37
GAPDH
C
Percent survival
100
50
0
months elapsed
0
5
10
15
20
flox
Δhep

Fig S2

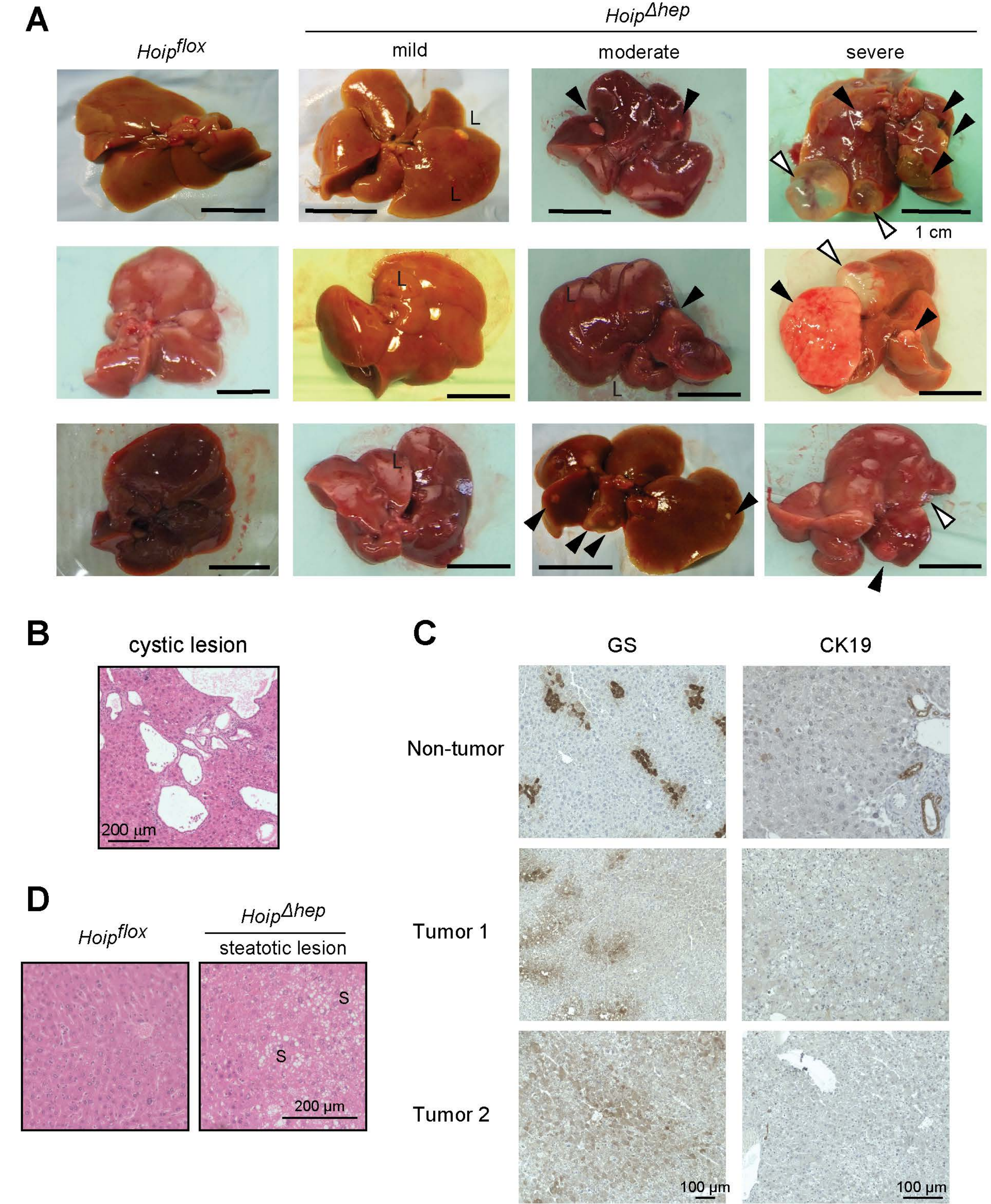
A
Hoipflox
HoipΔhep
mild
moderate
severe
L
1 cm
B
cystic lesion
200 µm
C
GS
CK19
Non-tumor
Tumor 1
Tumor 2
100 µm
100 µm
D
Hoipflox
HoipΔhep
steatotic lesion
S
200 µm

Fig S3

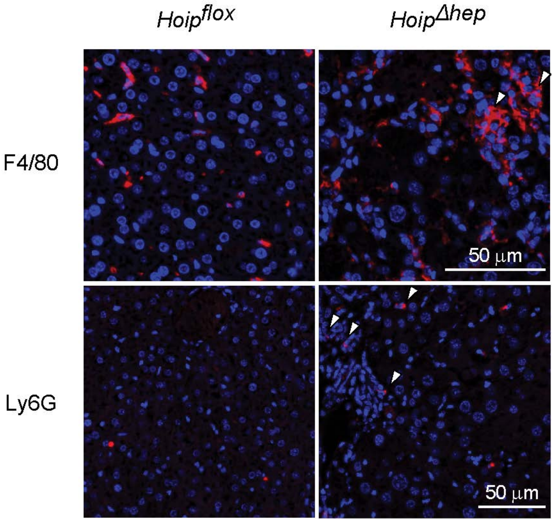

Fig S4

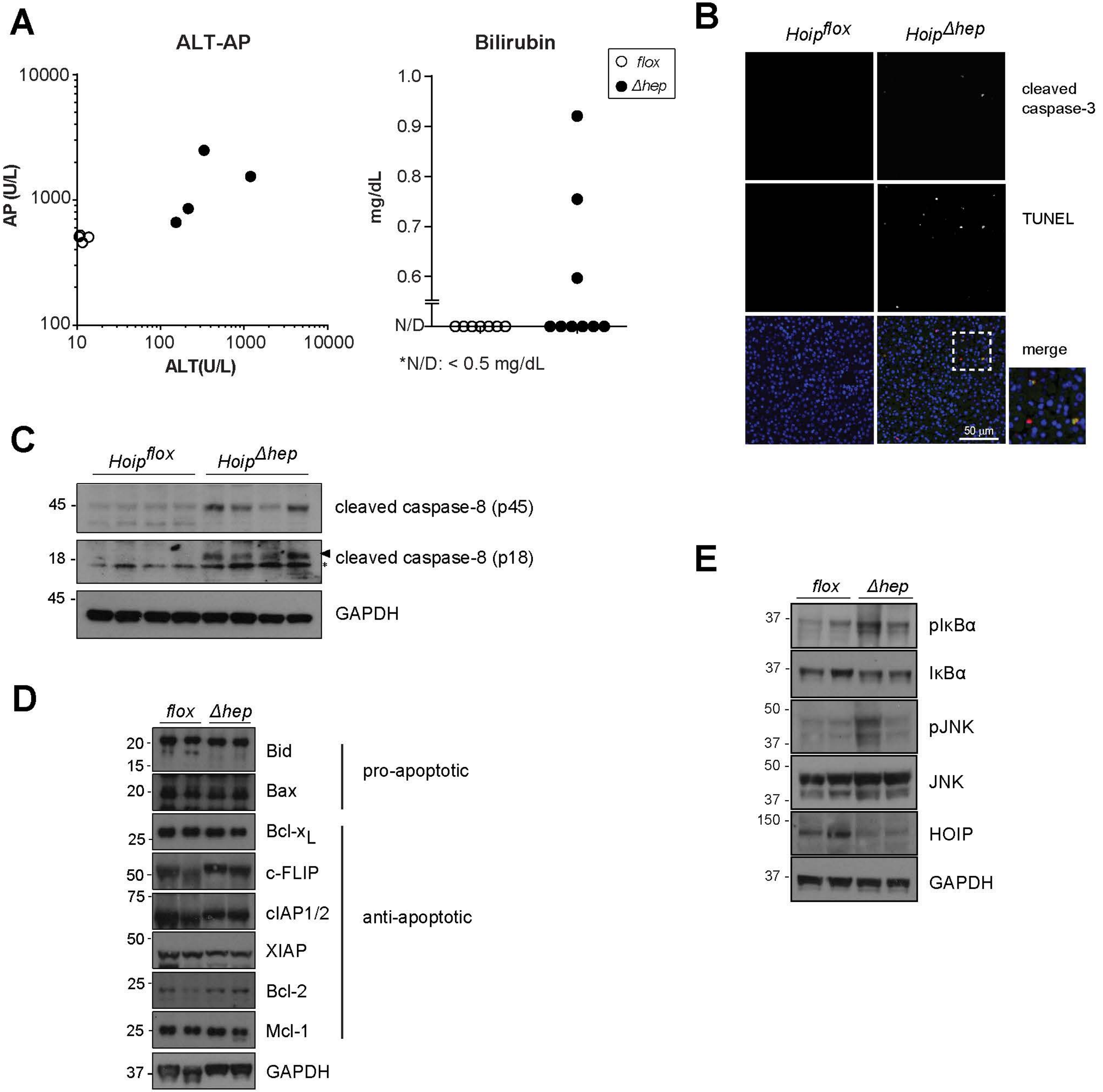
A
ALT-AP
10000
1000
100
AP (U/L)
10
100
1000
10000
ALT(U/L)
Bilirubin
flox
Δhep
1.0
0.9
0.8
0.7
0.6
N/D
mg/dL
*N/D: < 0.5 mg/dL
B
Hoipflox
HoipΔhep
cleaved caspase-3
TUNEL
merge
50 μm
C
Hoipflox
HoipΔhep
45
18
45
cleaved caspase-8 (p45)
cleaved caspase-8 (p18)
GAPDH
D
flox
Δhep
20
15
20
25
50
75
50
25
25
37
Bid
Bax
Bcl-xL
c-FLIP
cIAP1/2
XIAP
Bcl-2
Mcl-1
GAPDH
pro-apoptotic
anti-apoptotic
E
flox
Δhep
37
37
50
37
50
37
150
37
pIκBα
IκBα
pJNK
JNK
HOIP
GAPDH

Fig S5

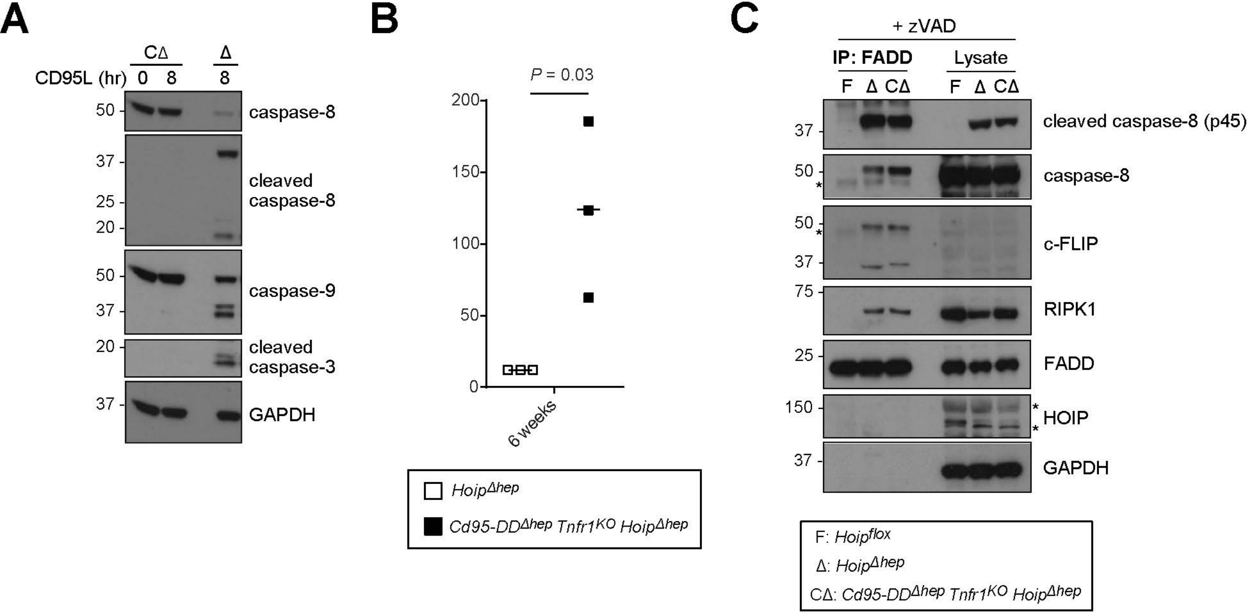

A
CΔ
Δ
CD95L (hr) 0 8 8
50
caspase-8
37
25
20
cleaved caspase-8
50
37
caspase-9
20
cleaved caspase-3
37
GAPDH
B
P = 0.03
200
150
100
50
0
6 weeks
Hoip^Δhep
Cd95-DD^Δhep Tnfr1^KO Hoip^Δhep
C
+ zVAD
IP: FADD
Lysate
F Δ CΔ F Δ CΔ
37
cleaved caspase-8 (p45)
50
caspase-8
50
37
c-FLIP
75
RIPK1
25
FADD
150
HOIP
37
GAPDH
F: Hoip^flox
Δ: Hoip^Δhep
CΔ: Cd95-DD^Δhep Tnfr1^KO Hoip^Δhep
